# Comparative Evaluation of Matrix- and Extraction-Dependent Phenolic Assay Responses in Selected Plant Materials

**DOI:** 10.3390/molecules31213993

**Published:** 2026-11-05

**Authors:** Mateusz Sugajski, Jacek Gajewski, Magdalena Buszewska-Forajta, Bogusław Buszewski

**Affiliations:** 1 Department of Environmental Chemistry and Bioanalytics, Faculty of Chemistry, Nicolaus Copernicus University in Toruń, Gagarina 7, 87-100 Toruń, Poland; m.sugajski@centrumczochralskiego.pl; 2 Prof. Jan Czochralski Kuyavian-Pomeranian Science and Technology Center in Toruń, Krasińskiego 4/4A, 87-100 Toruń, Poland; m.buszewska@umk.pl; 3 Faculty of Chemical and Process Engineering, Gdańsk University of Technology, Narutowicza 11/12, 80-233 Gdańsk, Poland; s189068@student.pg.edu.pl; 4 Institute of Veterinary Medicine, Faculty of Biological and Veterinary Sciences, Nicolaus Copernicus University in Toruń, Lwowska 1, 87-100 Toruń, Poland; 5 International Institute of Analytical Chemistry, Polish Academy of Sciences, Krasińskiego 4–4A, 87-100 Toruń, Poland

**Keywords:** *Berberis vulgaris*, aqueous ethanol extraction, phenolic acids, plant extracts, ultrasound-assisted extraction

## Abstract

Eight commercial lyophilised plant materials were extracted by maceration, shaking-assisted extraction, or ultrasound-assisted extraction using water, 70% ethanol, or 96% ethanol. Total phenolic content (TPC), total flavonoid content (TFC), total phenolic acid content (TPA), and vanillin-reactive tannins (TANs) were determined spectrophotometrically in three independent extractions per condition. Plant materials were compared across nine extraction systems, and the effects of extraction protocol, solvent system, and their interaction in barberry fruit were assessed by two-way ANOVA. Extraction behaviour was matrix- and response-dependent. Barberry fruit produced the highest TPC and TPA in all systems, whereas TFC and TAN rankings varied among conditions. Water and 70% ethanol generally produced higher assay responses than 96% ethanol. In barberry fruit, extraction protocol, solvent system, and their interaction affected TPC, TFC, and TPA, while the TAN value varied among solvent-defined systems, although cross-solvent comparisons may also reflect differences in colour development caused by the solvent composition of the vanillin reaction mixture. Aqueous ultrasound-assisted extraction produced the highest observed mean TFC, TPA, and TAN responses while retaining a comparatively high TPC response; however, no extraction condition was statistically superior across all four responses. These findings support barberry fruit as a model matrix for targeted chemical characterisation and further extraction studies.

## 1. Introduction

Plant materials contain chemically diverse phenolic constituents, including phenolic acids, flavonoids, and condensed-tannin-related compounds. Their extractability depends on the botanical matrix, plant organ, processing history, solvent composition, and extraction conditions. Consequently, results obtained for one plant material or extraction system cannot necessarily be transferred directly to another matrix.

Spectrophotometric assays are widely used for the preliminary comparison of plant extracts because they provide rapid operational measures of broad groups of reactive constituents. Total phenolic content (TPC), total flavonoid content (TFC), total phenolic acid content (TPA), and vanillin-reactive tannins (TANs) represent complementary assay-defined responses rather than complete or compound-specific compositional measurements. Differences among these responses may reflect both the chemical composition of the plant matrix and the selectivity of the analytical reaction.

The plant materials investigated in this study—barberry fruit and root (*Berberis vulgaris*), white mulberry fruit and leaves (*Morus alba*), hibiscus calyces (*Hibiscus sabdariffa*), ginger rhizome (*Zingiber officinale*), dandelion herb (*Taraxacum officinale*), and fenugreek seeds (*Trigonella foenum-graecum*)—were selected because they are used in traditional or contemporary plant-based preparations and have previously been investigated as sources of phenolic constituents [1,2,3,4,5,6]. Their traditional use provided the rationale for material selection but was not treated as evidence of biological efficacy in the present study.

Most extraction studies examine a single species, one plant organ, or a limited number of analytical endpoints. Differences between published studies therefore arise from both botanical variation and extraction design. A direct comparison conducted using a common solid-to-solvent ratio, the same solvent systems, defined extraction protocols, and identical analytical assays can help distinguish matrix-dependent patterns from differences associated with extraction conditions [7,8]. Related studies have likewise shown that solvent composition, extraction technique, and sample-treatment conditions can jointly shape polyphenolic profiles and measured assay responses [9,10].

Water, 70% ethanol, and 96% ethanol were selected to cover a broad range of solvent polarity. These solvent systems were used as comparative extraction media and were not intended to reproduce traditional infusion preparation. Similarly, maceration, shaking-assisted extraction, and ultrasound-assisted extraction were treated as complete extraction protocols rather than as isolated mechanical factors.

The objective of this study was to compare TPC, TFC, TPA, and TAN responses obtained from eight lyophilised plant materials using three extraction protocols and three solvent systems. The screening stage was designed to identify matrix- and assay-dependent patterns and to select a suitable plant material for subsequent targeted studies. Barberry fruit was then examined in greater detail to assess the effects of extraction protocol, solvent system, and their interaction on the four assay-defined responses. Individual phenolic compounds and biological activity were not evaluated. Accordingly, the present study was designed as a foundational comparative screening investigation. Its purpose was to identify matrix- and protocol-dependent differences in broad spectrophotometric responses and to provide a rational basis for selecting a botanical matrix and extraction conditions for subsequent targeted chromatographic and biological studies.

## 2. Results and Discussion

### 2.1. Comparative Screening Across Plant Materials

**Figure 1:**
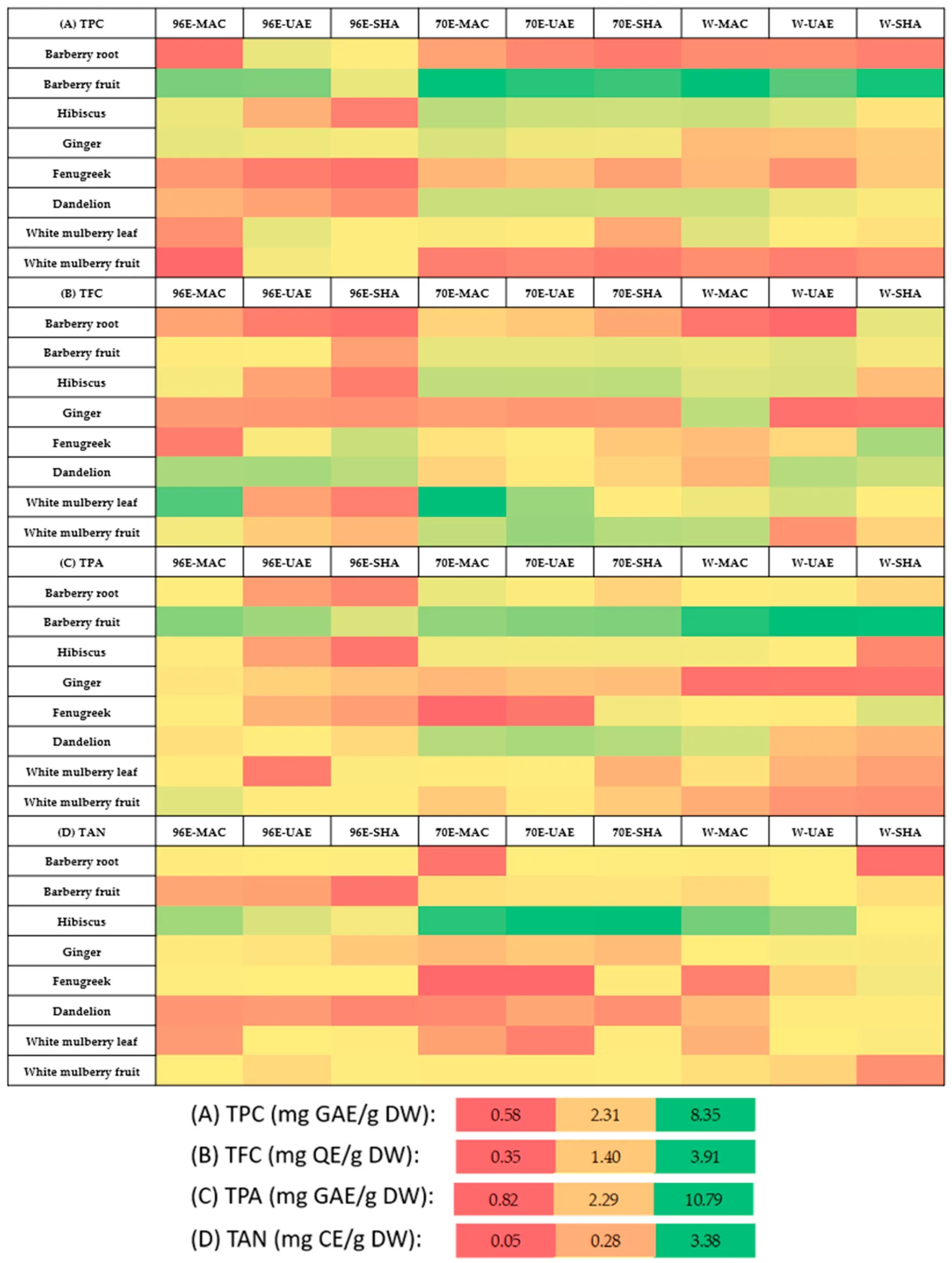
Heatmap representation of (**A**) total phenolic content (TPC); (**B**) total flavonoid content (TFC), (**C**) total phenolic acid content (TPA), and (**D**) vanillin-reactive tannins (TANs) in extracts obtained from eight plant materials using different extraction protocols and solvent systems. Cell colours represent the untransformed condition values calculated from the valid independent extractions available for each condition (*n* = 1–3); the data were plotted directly without normalisation or standardisation. Measurements outside the applicable calibration range were excluded. For conditions with *n* ≥ 2, the plotted value represents the mean, whereas for *n* = 1, the single valid result is shown. Because the four assays use different reference equivalents and numerical ranges, a separate continuous red–orange–green scale was applied to each panel. Red, orange, and green correspond to the minimum, 50th percentile, and maximum condition value, respectively, for the corresponding assay. The numerical scale anchors and assay-specific units are displayed below the heatmap. Colour intensity should therefore be compared within an individual panel and not between different assays. MAC, maceration; UAE, ultrasound-assisted extraction; SHA, shaking-assisted extraction; 96E, 96% ethanol; 70E, 70% ethanol; W, water.

The standardised screening design revealed substantial differences among plant materials and showed that solvent system and extraction protocol did not affect all matrices or assay responses in the same way (Table 1 and Table 2; Figure 1).

Barberry fruit produced the highest TPC response in every extraction system. Its values were highest in water and 70% ethanol and consistently lower in 96% ethanol. Hibiscus and dandelion showed a similar preference for water-containing systems, but their TPC responses remained below those of barberry fruit under the corresponding conditions (Figure 1). These findings identify barberry fruit as the leading material when total Folin–Ciocalteu response is the primary screening criterion.

This result is consistent with reports describing *Berberis vulgaris* fruit as phenolic-rich and sensitive to solvent composition [11,12]. Published solvent rankings vary, however, because the measured response depends on both the extraction system and the analytical endpoint. Direct studies of barberry show that aqueous extraction can produce extracts with substantial phenolic contents and practical relevance for food-compatible applications.

TFC followed a different pattern, with the highest observed values varying among plant materials and extraction conditions (Table 2). White mulberry leaves showed the highest mean TFC under maceration with 96% and 70% ethanol, whereas dandelion showed the highest means under UAE and SHA with 96% ethanol and under aqueous UAE. White mulberry fruit showed the highest mean TFC under 70% ethanol + UAE (2.77 mg QE/g DW, *n* = 2) and 70% ethanol + SHA (2.44 mg QE/g DW, *n* = 3). Under aqueous maceration, the highest observed value was also recorded for white mulberry fruit (2.35 mg QE/g DW), but it represented a single valid extraction (*n* = 1) and should therefore be interpreted cautiously. Fenugreek showed the highest mean under aqueous SHA. Hibiscus produced comparatively high TFC responses in 70% ethanol but did not show the highest mean under any tested condition. These rankings are descriptive and do not establish statistically significant differences among plant materials. Barberry fruit maintained comparatively high TFC responses in water and 70% ethanol; its selection for focused analysis was nevertheless based on its consistently highest TPC and TPA responses across extraction systems.

Previous studies have reported that flavonoid assay responses in white mulberry fruit and leaf extracts vary with solvent composition and fruit maturity [13]. Water-containing ethanol systems have also produced higher flavonoid assay responses than absolute ethanol in hibiscus extracts [14], while diluted ethanol has been favoured for flavonoid extraction from dandelion [15]. Fenugreek extracts likewise show differences in measured phenolic content across ethanol concentrations [7]. These reports provide context for the matrix- and extraction-dependent variation observed here, but do not establish the relative ranking of the materials under the present experimental conditions.

The divergence between TPC and TFC is chemically and analytically plausible. The Folin–Ciocalteu assay reflects a broad pool of reducing substances, whereas the aluminium chloride response depends on flavonoid structure. Barberry fruits contain a diverse phenolic profile [11,12], while leaf and aerial tissues such as white mulberry leaves and dandelion may respond more strongly in flavonoid-oriented assays [13,15]. The two indices should therefore be interpreted as complementary screening responses.

Across materials, water and 70% ethanol generally produced higher TPC and TFC responses than 96% ethanol, but the size of this advantage depended on the botanical matrix and extraction protocol. The absence of a uniform ranking supports a matrix-specific interpretation rather than selection of a universally superior solvent.

### 2.2. Distribution of Phenolic Acids and Vanillin-Reactive Tannins

TPA and TANs produced contrasting material rankings and further demonstrated that no single plant dominated every assay-defined response (Table 3 and Table 4).

Barberry fruit produced the highest TPA response in all nine extraction systems. Water gave the highest values, particularly with UAE and MAC, while 70% ethanol produced intermediate-to-high responses, and 96% ethanol was less effective. The consistency of this ranking across extraction protocols indicates that more polar extraction conditions produced higher Arnow-reactive responses in barberry fruit.

Chromatographic studies have identified gallic, chlorogenic, caffeic, and p-coumaric acids in *Berberis vulgaris* fruits [11,12], which makes the high TPA response chemically plausible. Direct quantitative comparison is not possible because the present Arnow assay is nonspecific and does not measure individual acids. The result should therefore be interpreted as a comparative assay response obtained within a common analytical system rather than as a compositional profile or a measure of absolute extraction recovery. The weak performance of 96% ethanol is nevertheless consistent with the limited extraction of highly polar phenolics by low-water solvent systems [7].

TAN showed a different distribution. Hibiscus produced the highest responses in most water and 70% ethanol systems, whereas fenugreek gave the maximum value under one aqueous shaking condition. Barberry fruit produced a measurable vanillin-reactive response but was not the leading material in the TAN assay. TAN was therefore more material-specific than TPC or TPA and was particularly sensitive to the interaction between matrix composition and solvent.

High tannin responses in hibiscus are consistent with studies of roselle calyces and hibiscus tea, where tannins are technologically relevant and may be deliberately reduced during beverage processing. Solvent-dependent differences have also been reported for hibiscus extracts [16,17]. In fenugreek, condensed-tannin measurements vary with cultivar and solvent, supporting the isolated high response observed under aqueous shaking [7,18].

The screening stage therefore identified two distinct findings. First, barberry fruit was the only material that combined the highest TPC and TPA responses across every extraction system. Second, TFC and TAN maxima shifted among matrices and conditions. The choice of a model material consequently depends on the analytical objective: hibiscus is particularly relevant for vanillin-reactive tannins, whereas barberry fruit offers the most consistent overall profile for TPC- and TPA-oriented work.

Barberry fruit was selected for focused analysis because its high responses were reproduced across all nine systems rather than being confined to one favourable condition. This criterion prioritised consistency and suitability for subsequent extraction studies over a claim of universal superiority across all phenolic groups.

This selection is supported by the documented chemical diversity of *Berberis vulgaris* fruits [11,12], variation in their phenolic and antioxidant profiles across genotypes and plant organs [12,19], and their sensitivity to harvest and processing conditions [20,21]. Application-oriented studies also support their use as a food-compatible source of phenolics and natural colourants [21]. Evidence concerning metabolic outcomes remains supportive but not definitive [1,22]; the present screening results therefore justify barberry fruit as a model matrix for further chemical and biological work, not as proof of clinical superiority.

Because the replicate extracts for each plant material were prepared from the same commercial product, the study evaluates extraction-level repeatability but does not account for biological or between-batch variability. The observed material-dependent patterns should therefore not be generalised to all commercial batches or geographic origins of the investigated species.

Figure 1 integrates the four assay responses and shows both the consistent TPC and TPA performance of barberry fruit and the condition-dependent distribution of TFC and TAN across the remaining materials.

### 2.3. Extraction Protocol and Solvent-System Effects in Barberry Fruit

**Figure 2:**
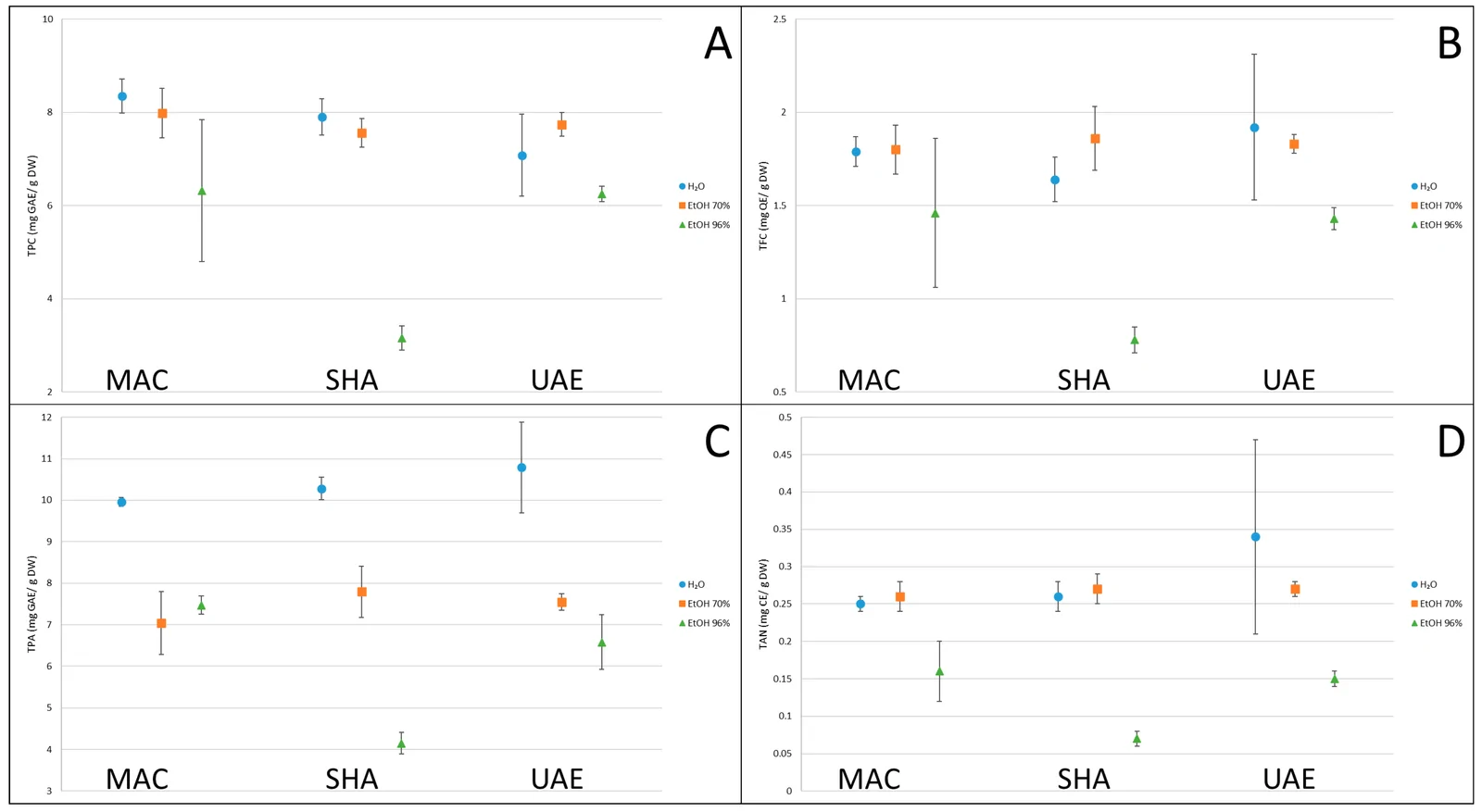
Effects of extraction protocol and solvent system on assay-defined phenolic responses in barberry fruit: (**A**) total phenolic content (TPC), (**B**) total flavonoid content (TFC), (**C**) total phenolic acid content (TPA), and (**D**) vanillin-reactive tannins (TANs). Symbols represent mean values, and error bars indicate standard deviations from three independently prepared extracts (*n* = 3). Different symbols and colours denote water, 70% (*v*/*v*) ethanol, and 96% (*v*/*v*) ethanol. MAC, maceration; SHA, shaking-assisted extraction; UAE, ultrasound-assisted extraction.

Two-way ANOVA was used to examine the effects of extraction protocol, solvent system, and their interaction in barberry fruit, followed by Tukey HSD comparisons among the nine complete protocol–solvent combinations (Table 5, Figure 2). The relative importance of these factors differed among assay responses, and the post hoc comparisons did not identify one extraction condition that was statistically superior across all four responses.

TPC was significantly affected by extraction protocol (F = 9.54, *p* = 0.0015), solvent system (F = 44.20, *p* < 0.0001), and their interaction (F = 8.11, *p* = 0.0006). H_2_O + MAC produced the highest mean TPC response (8.35 mg GAE/g DW). Among the remaining conditions, 70% ethanol + MAC produced a slightly higher mean TPC response (7.98 mg GAE/g DW) than H_2_O + SHA (7.90 mg GAE/g DW). However, Tukey’s HSD test showed that H_2_O + MAC did not differ significantly from the other aqueous protocols or from any of the 70% ethanol protocols (adjusted *p* > 0.05). It produced significantly higher responses than all three 96% ethanol conditions. The lowest TPC response was obtained with 96% ethanol + SHA (3.16 mg GAE/g DW).

TFC was significantly affected by extraction protocol (F = 5.60, *p* = 0.0128), solvent system (F = 23.41, *p* < 0.0001), and their interaction (F = 3.05, *p* = 0.0439). H_2_O + UAE produced the highest mean response (1.92 mg QE/g DW), but it did not differ significantly from the remaining aqueous conditions or from any of the 70% ethanol conditions. Among the comparisons involving H_2_O + UAE, a significant difference was observed only relative to 96% ethanol + SHA. The highest observed TFC mean should therefore not be interpreted as evidence of the general statistical superiority of aqueous UAE.

TPA was significantly affected by extraction protocol (F = 6.64, *p* = 0.0069), solvent system (F = 136.28, *p* < 0.0001), and their interaction (F = 12.33, *p* < 0.0001). H_2_O + UAE produced the highest mean response (10.79 mg GAE/g DW). Its response was significantly higher than those obtained using all six ethanolic conditions, but it did not differ significantly from H_2_O + MAC (9.96 mg GAE/g DW) or H_2_O + SHA (10.28 mg GAE/g DW). These results support an advantage of the aqueous extraction systems for the Arnow-reactive response but do not establish a specific advantage of UAE over the other aqueous protocols.

For TAN, solvent system was the only significant factor (F = 28.56, *p* < 0.0001), whereas extraction protocol (F = 3.00, *p* = 0.0754) and the protocol × solvent-system interaction (F = 1.91, *p* = 0.1533) were not significant. H_2_O + UAE produced the highest mean TAN response (0.34 mg CE/g DW), but it did not differ significantly from the other aqueous conditions or from the 70% ethanol conditions. It differed significantly only from the three 96% ethanol conditions. Accordingly, the observed TAN responses differed among solvent-defined systems and did not demonstrate a UAE-specific effect. However, because the reaction-mixture solvent composition was not matched among aqueous and ethanolic extracts, the cross-solvent differences cannot be attributed exclusively to extraction.

Taken together, the post hoc comparisons did not identify H_2_O + UAE as a statistically superior multi-response optimum. This condition was retained as a practical candidate for follow-up studies because it combined water as the extraction solvent with high mean TFC, TPA, and TAN responses and a comparatively high TPC response. Its selection should therefore be understood as a pragmatic, response-balanced choice within the tested design rather than as the outcome of a formal multi-response optimisation.

### 2.4. Descriptive Relationships Among Assay Responses

Pearson correlation coefficients were recalculated using pairwise complete condition means after excluding assay–condition values based on only one valid independent extraction (*n* = 1). Depending on the pair of assays, the analysis included 67–72 paired condition means (Table 6). Because these observations originated from a structured experimental design and included repeated combinations within the same botanical matrices, the coefficients were treated exclusively as descriptive measures. They do not represent associations among independently sampled biological observations and cannot distinguish matrix-related effects from those associated with extraction conditions.

The strongest descriptive relationship was observed between TPC and TPA (r = 0.84; *n* = 70), indicating that conditions producing higher Folin–Ciocalteu responses also generally produced higher Arnow assay responses. Both assays are nonspecific and were applied to the same extracts; therefore, this relationship does not demonstrate that phenolic acids quantitatively account for the measured TPC response.

The remaining coefficients indicated only weak descriptive co-variation. TFC was weakly associated with TANs (r = 0.20; *n* = 69), TPAs (r = 0.12; *n* = 67), and TPC (r = 0.07; *n* = 69). A weak positive coefficient was also observed between TPC and TAN (r = 0.15; *n* = 72). These results support the interpretation that the four assays provide related but non-equivalent information and respond differently to botanical matrix composition and extraction conditions. Differences in the relationships among phenolic-related assay responses have also been reported for other botanical matrices [23].

The coefficient between the TPA and TAN responses was close to zero and slightly negative (r = −0.04; *n* = 70). Thus, within the investigated combinations, the vanillin-reactive response varied largely independently of the Arnow-reactive response. Comparisons with studies using different tannin assays should account for the selective response of the vanillin method [24].

Extract-specific blanks prepared without the respective colour-forming reagents were not measured. Although the diluted extracts did not exhibit pronounced visible colouration and no anomalous absorbance patterns were observed among the replicate measurements, a contribution of intrinsic matrix absorbance to the signals recorded at 428, 500, and 525 nm cannot be completely excluded. The TFC, TPA, and TAN results should therefore be interpreted as apparent, assay-defined responses obtained under the applied analytical conditions rather than as selective measurements of individual compound classes.

A further limitation applies to comparisons of TAN responses among solvent systems. The solvent composition of the catechin standards was not matched separately to that of the aqueous and hydroethanolic extracts. Because water content can affect colour development in the vanillin reaction, the differences observed among water, 70% ethanol, and 96% ethanol systems may reflect both extraction-related differences and solvent-dependent variation in analytical response. Cross-solvent TAN comparisons should therefore not be interpreted as isolated effects of solvent on tannin extraction. Comparisons among plant materials or extraction protocols within the same solvent system remain less affected by this source of analytical variation because the reaction-mixture solvent composition was consistent within those comparisons. Future studies should employ solvent-matched calibration or standard-addition procedures to minimise solvent-dependent variation in colour development.

Taken together, these descriptive coefficients illustrate that the four spectrophotometric assays provide partly overlapping but non-equivalent information about the investigated extracts. The Folin–Ciocalteu assay reflects overall reducing capacity, the aluminium chloride response depends on flavonoid structure, and the Arnow and vanillin assays may respond selectively to different, partially overlapping groups of constituents in complex extracts [24,25,26,27]. Chromatographic approaches are therefore required for the selective determination of individual phenolic acids and flavonoids [28].

Moreover, the spectrophotometric assays provide operational indices of broad groups of reactive constituents rather than compound-specific measurements. Although three independent extracts were prepared for every condition, measurements outside the applicable calibration ranges were excluded, and some screening summaries were consequently based on fewer than three valid results. The small number of independent extracts and the absence of formal assessment of model assumptions require cautious interpretation of the inferential results. Nevertheless, the consistent performance of barberry fruit across extraction systems provides a basis for its selection as a model matrix for subsequent chromatographic characterisation and biological testing.

## 3. Materials and Methods

### 3.1. Plant Materials

Eight commercially available lyophilised plant materials were studied: barberry fruit and root (*Berberis vulgaris*), white mulberry fruit and leaves (*Morus alba*), hibiscus calyces (*Hibiscus sabdariffa*), ginger rhizome (*Zingiber officinale*), dandelion herb (*Taraxacum officinale*), and fenugreek seeds (*Trigonella foenum-graecum* L.). The materials were purchased from the online retailer liofilizaty.com (**Busko-Zdrój, Poland**) and stored in their original, tightly closed packages in a dry, dark place at 20 ± 2 °C. Because the products were obtained as commercial lyophilisates, batch-specific identifiers, exact geographic origin, voucher specimens or independent botanical-authentication records, particle-size specifications, and moisture measurements were not available. Results were calculated per gram of lyophilised material. For each plant material, the three independent extracts were prepared from separate portions (approximately 0.5 g each) taken from the same purchased commercial lyophilised product; different production batches were not used.

### 3.2. Extraction of Bioactive Compounds

Each material was extracted by maceration (MAC), shaking-assisted extraction (SHA), and ultrasound-assisted extraction (UAE) [29]. All samples were protected from direct light during extraction.

- Maceration (MAC): samples were kept in closed vessels for 24 h at 50 °C in a W615E thermostated water bath (Laboplay, Bytom, Poland).
- Shaking-assisted extraction (SHA): samples were shaken for 30 min at 200 rpm at room temperature using an EL680Q laboratory shaker (Eberbach, Belleville, MI, USA).
- Ultrasound-assisted extraction (UAE) was performed for 30 min in tightly closed 50 mL vessels using an Elmasonic Select 60 ultrasonic bath (Elma Schmidbauer GmbH, Singen, Germany), operating at a nominal ultrasonic frequency of 37 kHz and a nominal effective ultrasonic power of 150 W. The ultrasonic power was not adjustable during the experiment. These values represent the manufacturer’s specifications; the acoustic power delivered directly to the sample was not measured independently. No external temperature control was applied, and the bath temperature was not monitored during extraction.

Because extraction time and temperature differed among MAC, SHA, and UAE, these treatments were regarded as complete extraction protocols rather than as experiments isolating the individual effects of maceration, mechanical agitation, or ultrasound. All extractions were initiated with 20 mL of solvent and performed in tightly closed 50 mL vessels to minimise solvent loss. No visible solvent loss was observed; therefore, the extracts were not adjusted to a final volume after extraction. The initial nominal extraction volume of 20 mL was used in the calculation of the assay responses expressed per gram of dry material.

Distilled water and 96% (*v*/*v*) ethanol (Honeywell, Charlotte, NC, USA) were used as extraction solvents. The 70% (*v*/*v*) ethanol solution was prepared by diluting 96% ethanol with distilled water. In every extraction, approximately 0.5 g of lyophilised material was combined with 20 mL of solvent, corresponding to a nominal solid-to-solvent ratio of 1:40 (*m*/*v*). The exact sample mass was recorded for each independent extraction and used in the calculation of the assay responses expressed per gram of lyophilised material.

After extraction, samples were centrifuged at 5000 rpm for 10 min using a Multi FC5707 laboratory centrifuge equipped with an R05 rotor (OHAUS, Parsippany, NJ, USA). Supernatants were collected and stored at −25 °C until analysis. Three independent extracts were prepared for each combination of plant material, extraction protocol, and solvent system (*n* = 3 independent extractions).

### 3.3. Determination of Bioactive Compounds

Folin–Ciocalteu reagent, sodium carbonate, aluminium chloride, acetic acid, sodium nitrite, sodium molybdate, hydrochloric acid, sodium hydroxide, vanillin, sulphuric acid, and methanol were obtained from Chempur (Piekary Śląskie, Poland). Gallic acid was obtained from Pol-Aura (Morąg, Poland), whereas quercetin and catechin were obtained from Sigma-Aldrich (St. Louis, MO, USA).

Spectrophotometric measurements were performed using a BK-UV 1800PC UV–Vis spectrophotometer (Biobase, Jinan, China) and standard quartz cuvettes with a 1 cm optical path length and a nominal volume of 4 mL. Four assay-defined responses were measured: Folin–Ciocalteu-reactive substances (reported as TPC), aluminium-chloride-reactive flavonoids (TFC), Arnow-reactive phenolic acids (TPAs), and vanillin-reactive tannins (TANs). These assays provide comparative indices and do not represent exhaustive or chemically specific measurements of each compound class. Because no exhaustive reference extraction or mass-balance analysis was performed, the reported values represent assay-defined contents in the obtained extracts and should not be interpreted as absolute extraction recoveries or recovery efficiencies.

TPC was determined using the Folin–Ciocalteu assay. An aliquot of extract (0.25 mL) was mixed with 0.25 mL of Folin–Ciocalteu reagent in a 5 mL volumetric flask. After 5 min, 0.5 mL of sodium carbonate solution (120 g/L) was added, and the mixture was diluted to volume with distilled water. After incubation for 30 min at room temperature in the dark, absorbance was measured at 725 nm against the assay-specific zero-standard reagent blank. The gallic acid calibration range was 10–250 mg/L, and the relationship between absorbance (A) and standard concentration (c, mg/L) was A = 0.003747c–0.013822 (R^2^ = 0.9984). Results were expressed as mg gallic acid equivalents per gram of lyophilised material (mg GAE/g DW) [25].

TFC was measured using aluminium chloride complexation. Diluted extract (0.4 mL) was mixed with 0.6 mL of AlCl_3_ solution (20 g/L) and made up to 5 mL with a diluent prepared by mixing 80% (*v*/*v*) acetic acid with ethanol at a ratio of 1:19 (*v*/*v*), corresponding to a final acetic acid concentration of 4% (*v*/*v*) in the diluent. After 30 min at room temperature, absorbance was measured at 428 nm against the assay-specific zero-standard reagent blank. The quercetin calibration range was 10–200 mg/L, and the relationship between absorbance (A) and standard concentration (c, mg/L) was A = 0.005119c–0.003250 (R^2^ = 0.9999). Results were expressed as mg quercetin equivalents per gram of lyophilised material (mg QE/g DW) [26].

TPA was estimated using the Arnow assay. Extract (0.3 mL) was reacted sequentially with Arnow reagent, hydrochloric acid, and sodium hydroxide as described previously [27]. Absorbance was measured at 525 nm against the assay-specific zero-standard reagent blank. The gallic acid calibration range was 20–160 mg/L, and the relationship between blank-corrected absorbance (A) and standard concentration (c, mg/L) was A = 0.0052c–0.0407 (R^2^ = 0.9980). Results were expressed as mg gallic acid equivalents per gram of lyophilised material (mg GAE/g DW).

TAN was estimated using the vanillin assay. Extract (0.5 mL) was mixed with 1.0 mL of 1% (*m*/*v*) vanillin solution in ethanol and 1.25 mL of 4.5 M sulphuric acid solution in methanol, and the reaction mixture was subsequently made up to 5 mL with ethanol. After incubation for 15 min at 30 °C, absorbance was measured at 500 nm against the assay-specific zero-standard reagent blank. The catechin calibration range was 1.175–11.750 mg/L, and the relationship between blank-corrected absorbance (A) and standard concentration (c, mg/L) was A = 0.0114c + 0.0001 (R^2^ = 0.9954). Results were expressed as mg catechin equivalents per gram of lyophilised material (mg CE/g DW) [24]. Because the vanillin reaction preferentially detects selected flavan-3-ols and condensed-tannin-related structures, TAN was treated as a comparative response rather than total tannin content. Catechin calibration solutions were prepared in ethanol. The solvent composition of the standards was not separately matched to that of the aqueous, 70% ethanol, and 96% ethanol extracts. Because 0.5 mL of extract was added to a final reaction volume of 5 mL, the approximate contribution of water originating from the sample was 10%, 3%, and 0.4% (*v*/*v*) for the aqueous, 70% ethanol, and 96% ethanol extracts, respectively.

The stated calibration ranges refer to the concentrations of the working standard solutions before their addition to the assay reaction mixtures. Because the assay responses differed among plant materials and extraction conditions, the extracts were individually diluted with the corresponding extraction solvent, when required, until the measured response fell within the applicable calibration range. No single dilution scheme was therefore applied to the complete sample set. Measurements outside the calibration range were not used for quantitative calculations.

The assay responses were calculated according to X = (c × d × V)/m, where X is expressed as mg standard equivalents per gram of dry material, c is the concentration obtained from the calibration equation for the diluted extract (mg/L), d is the dilution factor, V is the extraction volume (0.020 L), and m is the mass of lyophilised plant material used for extraction (g). The replicate-level calculation audit, including sample masses, extraction volumes, absorbance values, calibration parameters, dilution corrections, calibration-range verification, back-calculated extract concentrations, and final assay results, is provided in Supplementary File S2.

For each assay, an assay-specific zero-standard reagent blank was prepared using the same reagent system as that employed for calibration but without an analytical standard or plant extract. The TPC blank contained Folin–Ciocalteu reagent, sodium carbonate, and distilled water; the TFC blank contained aluminium chloride and the acetic acid–ethanol diluent; the TPA blank contained Arnow reagent, hydrochloric acid, and sodium hydroxide; and the TAN blank contained vanillin, sulphuric acid in methanol, and ethanol. A single assay-specific reagent blank was used for all extracts irrespective of the extraction solvent. Separate blanks matched to water, 70% ethanol, and 96% ethanol were not prepared. Extract-specific blanks containing the extract but lacking the respective colour-forming reagent were also not measured; consequently, the intrinsic absorbance of the extracts was not separately subtracted from the analytical signal.

### 3.4. Statistical Analysis

The experimental unit was an independently prepared extract. Three independent extracts were prepared for each plant material × extraction protocol × solvent-system combination. Measurements outside the applicable calibration range were excluded from quantitative analysis. Consequently, the comparative screening summaries were based on one to three valid independent measurements per assay–condition combination, with the applicable *n* indicated in Table 1, Table 2, Table 3 and Table 4. Standard deviation was not calculated when only one valid result remained. All barberry fruit conditions retained three valid independent results and were included in the inferential analysis. Technical measurements from the same extract were averaged before statistical analysis. Statistical significance was set at α = 0.05. Formal tests of residual normality and homogeneity of variance were not performed. Given the small number of independent extracts per condition, inferential results were interpreted cautiously.

Plant materials were compared descriptively within each of the nine extraction systems using mean values and standard deviations. Because the screening stage was intended to identify consistent matrix-dependent patterns, no inferential pairwise comparisons among plant materials were retained.

For barberry fruit, a two-way ANOVA was fitted separately to each response, with extraction protocol (MAC, SHA, and UAE), solvent system (water, 70% ethanol, and 96% ethanol), and the protocol × solvent-system interaction as fixed effects. Pairwise comparisons among the nine complete protocol–solvent combinations were subsequently performed using Tukey’s honestly significant difference test, with multiplicity-adjusted *p*-values. Condition-level comparisons were used because significant interaction effects indicated that the influence of the extraction protocol depended on the solvent system for several responses. Statistical significance was established at *p* < 0.05. The individual extraction-level data, condition-level descriptive statistics, complete two-way ANOVA tables, and Tukey HSD pairwise comparisons are provided in Supplementary File S1.

Pearson correlation coefficients were calculated as descriptive measures of co-variation among the four assay responses using pairwise complete condition means. Assay–condition values based on only one valid independent extraction (*n* = 1) were excluded from the correlation analysis. Consequently, the number of paired observations varied from 67 to 72, depending on the pair of assays. Because the condition means originated from a structured experimental design and did not represent independently sampled biological observations, no inferential interpretation was assigned to the correlation coefficients, and correlation *p*-values were not reported. Calculations were performed in Microsoft Excel (Microsoft, Redmond, WA, USA).

## 4. Conclusions

The present study’s results provide a methodological foundation for a broader sequence of investigations involving targeted chromatographic characterisation, optimisation of extraction conditions, and assessment of the biological properties of selected extracts.

The standardised comparison demonstrated that the four spectrophotometric responses depended on the botanical matrix, solvent system, extraction protocol, and analytical assay. Barberry fruit produced the highest TPC and TPA responses across the tested extraction systems, whereas the materials producing the highest TFC and TAN responses varied among conditions. No plant material or extraction protocol was therefore uniformly superior across all four assays.

Water and 70% ethanol generally produced higher assay responses than 96% ethanol. For TAN, however, cross-solvent differences should be interpreted cautiously because the solvent composition of the catechin standards was not matched to that of the extracts and may have affected colour development in the vanillin reaction. In barberry fruit, aqueous maceration produced the highest mean TPC response, whereas aqueous ultrasound-assisted extraction produced the highest mean TFC, TPA, and TAN responses. However, Tukey’s HSD comparisons showed that aqueous ultrasound-assisted extraction was not significantly superior to all alternative extraction conditions across these responses. These maxima should therefore be interpreted as descriptive results within the tested design rather than as evidence of the overall statistical superiority of this extraction condition.

Barberry fruit can therefore be considered a suitable model matrix for subsequent targeted chromatographic characterisation, extraction-method development, and biological evaluation. In this context, the present comparative screening study constitutes the methodological foundation for the next stages of the research rather than a comprehensive characterisation of the investigated materials. Because the present study used broad spectrophotometric indices and did not include biological assays, no conclusions can be drawn regarding the concentrations of individual compounds or the biological and antidiabetic activity of the investigated extracts.

## Figures and Tables

**Table 1:** Total phenolic content (TPC) under each extraction condition. Values are presented as mean ± sample standard deviation. Measurements outside the applicable calibration range were excluded. The number of valid independent extractions is indicated where *n* < 3; for *n* = 1, only the individual valid result is reported and SD is not applicable.

	EtOH 96% + MAC [mg/g DW]	EtOH 96% + UAE [mg/g DW]	EtOH 96% + SHA [mg/g DW]	EtOH 70% + MAC [mg/g DW]	EtOH 70% + UAE [mg/g DW]	EtOH 70% + SHA [mg/g DW]	H_2_O + MAC [mg/g DW]	H_2_O + UAE [mg/g DW]	H_2_O + SHA [mg/g DW]
Barberry root	0.70 ± 0.05	3.21 ± 0.13	2.38 ± 0.79	1.34 ± 0.08	0.97 ± 0.09	0.80 ± 0.10	1.04 ± 0.02	1.05 ± 0.05	0.88 ± 0.13
Barberry fruit	6.32 ± 1.52	6.25 ± 0.16	3.16 ± 0.26	7.98 ± 0.53	7.74 ± 0.25	7.56 ± 0.31	8.35 ± 0.37	7.08 ± 0.88	7.90 ± 0.39
Hibiscus	3.15 ± 0.15	1.51 ± 0.28	0.88 ± 0.03	4.61 ± 0.64	4.10 ± 0.17	4.05 ± 0.04	4.24 ± 0.38	3.68 ± 0.33	2.21 ± 0.27
Ginger	3.28 ± 0.34	3.05 ± 0.04	2.81 ± 0.09	3.74 ± 0.48	3.11 ± 0.07	2.98 ± 0.34	1.66 ± 0.57	1.72 ± 0.27	1.89 ± 0.09
Fenugreek	1.18 ± 0.03	0.84 ± 0.05	0.69 ± 0.04	1.61 ± 0.19	1.74 ± 0.08	1.30 ± 0.02	1.63 ± 0.10	1.13 ± 0.10	1.86 ± 0.15
Dandelion	1.57 ± 0.13	1.34 ± 0.04	1.07 ± 0.02	4.23 ± 0.67	4.24 ± 0.18	4.11 ± 0.12	4.15 ± 0.29	3.12 ± 0.12	2.73 ± 0.26
White mulberry leaf	1.09 ± 0.15	3.33 ± 0.55	2.35 ± 0.08	2.72 ± 0.18	2.30 ± 0.27	1.39 ± 0.16	3.53 ± 0.44	2.39 ± 0.32	2.16 ± 0.06
White mulberry fruit	0.58 ± 0.03	2.91 ± 0.44	2.31 ± 0.05	0.86 ± 0.03	0.95 ± 0.03	0.83 ± 0.04	1.06 ± 0.05	0.87 ± 0.11	1.02 ± 0.29

**Table 2:** Total flavonoid content (TFC) under each extraction condition. Values are presented as mean ± sample standard deviation. Measurements outside the applicable calibration range were excluded. The number of valid independent extractions is indicated where *n* < 3; for *n* = 1, only the individual valid result is reported and SD is not applicable. * Value calculated from two valid independent extractions (*n* = 2). ** Value represents a single valid independent extraction (*n* = 1); therefore, SD is reported as not applicable (N/A).

	EtOH 96% + MAC [mg/g DW]	EtOH 96% + UAE [mg/g DW]	EtOH 96% + SHA [mg/g DW]	EtOH 70% + MAC [mg/g DW]	EtOH 70% + UAE [mg/g DW]	EtOH 70% + SHA [mg/g DW]	H_2_O + MAC [mg/g DW]	H_2_O + UAE [mg/g DW]	H_2_O + SHA [mg/g DW]
Barberry root	0.82 ± 0.06	0.51 ± 0.11	0.42 ± 0.10 *	1.20 ± 0.05	1.10 ± 0.13	0.85 ± 0.10	0.45 ± 0.01 *	0.35 **	1.80 ± 0.04 *
Barberry fruit	1.46 ± 0.40	1.43 ± 0.06	0.78 ± 0.07	1.80 ± 0.13	1.83 ± 0.05	1.86 ± 0.17	1.79 ± 0.08	1.92 ± 0.39	1.64 ± 0.12
Hibiscus	1.60 ± 0.02	0.81 ± 0.13	0.51 ± 0.02	2.29 ± 0.36	2.27 ± 0.07	2.34 ± 0.04	1.92 ± 0.17	1.98 ± 0.12	1.03 ± 0.06
Ginger	0.74 ± 0.01	0.72 ± 0.04	0.69 ± 0.03	0.77 ± 0.03	0.72 ± 0.01	0.73 ± 0.02	2.32 ± 0.60 *	0.41 **	0.45 ± 0.01 *
Fenugreek	0.51 ± 0.02	1.57 ± 1.75 *	2.18 ± 0.07	1.33 ± 0.08	1.40 ± 0.02	1.11 ± 0.04	1.03 ± 0.01	1.23 ± 0.17	2.60 ± 0.15
Dandelion	2.54 ± 0.18	2.60 ± 0.07	2.35 ± 0.08	1.20 ± 0.07	1.37 ± 0.05	1.19 ± 0.03	0.96 ± 0.18	2.43 ± 0.05	2.19 ± 0.08
White mulberry leaf	3.43 ± 0.12	0.80 ± 0.14	0.53 ± 0.04	3.91 ± 0.19	2.73 ± 0.30	1.39 ± 0.19	1.73 ± 0.13	2.06 ± 0.23	1.41 ± 0.11
White mulberry fruit	1.66 ± 0.08	1.14 ± 0.20	0.98 ± 0.03	2.25 ± 0.18	2.77 ± 0.04 *	2.44 ± 0.06	2.35 **	0.68 ± 0.02	1.20 ± 1.43

**Table 3:** Total phenolic acid content (TPA) under each extraction condition. Values are presented as mean ± sample standard deviation. Measurements outside the applicable calibration range were excluded. The number of valid independent extractions is indicated where *n* < 3; for *n* = 1, only the individual valid result is reported and SD is not applicable. * Value calculated from two valid independent extractions (*n* = 2). ** Value represents a single valid independent extraction (*n* = 1); therefore, SD is reported as not applicable (N/A).

	EtOH 96% + MAC [mg/g DW]	EtOH 96% + UAE [mg/g DW]	EtOH 96% + SHA [mg/g DW]	EtOH 70% + MAC [mg/g DW]	EtOH 70% + UAE [mg/g DW]	EtOH 70% + SHA [mg/g DW]	H_2_O + MAC [mg/g DW]	H_2_O + UAE [mg/g DW]	H_2_O + SHA [mg/g DW]
Barberry root	2.43 ± 1.40	1.40 ± 0.13	1.15 ± 0.49 *	3.52 ± 0.08	2.75 ± 0.47	2.00 ± 0.21	2.50 ± 0.12	2.77 ± 0.07	2.04 ± 0.44
Barberry fruit	7.47 ± 0.22	6.58 ± 0.66	4.15 ± 0.26	7.04 ± 0.75	7.54 ± 0.20	7.79 ± 0.62	9.96 ± 0.11	10.79 ± 1.10	10.28 ± 0.27
Hibiscus	2.72 ± 0.12	1.43 ± 0.18	0.96 ± 0.04	3.17 ± 0.42	3.03 ± 0.07	3.18 ± 0.06	3.02 ± 0.21	2.37 ± 0.97	1.17 ± 0.22
Ginger	2.21 ± 0.13	1.99 ± 0.20	1.83 ± 0.06	1.72 ± 0.14	1.81 ± 0.03	1.78 ± 0.08	0.91 ± 0.00	0.92 ± 0.10	0.94 ± 0.04
Fenugreek	2.38 ± 0.24	1.64 ± 0.05	1.42 ± 0.06	0.82 ± 0.03 *	0.97 ± 0.02	3.13 ± 2.11	2.34 ± 0.07	2.67 ± 0.47	4.23 ± 0.56
Dandelion	2.15 ± 0.11	2.31 ± 0.12	2.07 ± 0.14	5.72 ± 0.29	6.21 ± 0.35	5.75 ± 0.29	4.46 ± 0.35	1.79 ± 0.11	1.65 ± 0.01
White mulberry leaf	2.28 ± 0.20	1.04 **	2.74 ± 1.52	2.55 ± 0.16	2.36 ± 0.22	1.64 ± 0.15	2.18 ± 0.60	1.65 ± 0.29	1.43 ± 0.03
White mulberry fruit	3.94 **	2.53 ± 1.57	2.49 ± 0.16	1.91 ± 1.81	2.26 ± 2.43	1.92 ± 1.45	1.58 ± 0.05	1.31 ± 0.15	1.24 ± 0.39 *

**Table 4:** Vanillin-reactive tannins (TANs) under each extraction condition. Values are presented as mean ± sample standard deviation. Measurements outside the applicable calibration range were excluded. The number of valid independent extractions is indicated where *n* < 3; for *n* = 1, only the individual valid result is reported and SD is not applicable. * Value calculated from two valid independent extractions (*n* = 2).

	EtOH 96% + MAC [mg/g DW]	EtOH 96% + UAE [mg/g DW]	EtOH 96% + SHA [mg/g DW]	EtOH 70% + MAC [mg/g DW]	EtOH 70% + UAE [mg/g DW]	EtOH 70% + SHA [mg/g DW]	H_2_O + MAC [mg/g DW]	H_2_O + UAE [mg/g DW]	H_2_O + SHA [mg/g DW]
Barberry root	0.37 ± 0.04	0.32 ± 0.08	0.35 ± 0.00 *	0.07 ± 0.00	0.29 ± 0.22	0.36 ± 0.05 *	0.38 ± 0.05	0.33 ± 0.02 *	0.06 ± 0.02 *
Barberry fruit	0.16 ± 0.04	0.15 ± 0.01	0.07 ± 0.01	0.26 ± 0.02	0.27 ± 0.01	0.27 ± 0.02	0.25 ± 0.01	0.34 ± 0.13	0.26 ± 0.02
Hibiscus	1.82 ± 0.02	1.01 ± 0.11	0.58 ± 0.02	3.06 ± 0.49	3.32 ± 0.18	3.38 ± 0.08 *	2.41 ± 0.28	2.03 ± 1.52	0.29 ± 0.03
Ginger	0.28 ± 0.02	0.27 ± 0.02	0.22 ± 0.01	0.20 ± 0.01	0.22 ± 0.01	0.20 ± 0.01	0.29 ± 0.18	0.49 ± 0.04	0.48 ± 0.02
Fenugreek	0.36 ± 0.02	0.30 ± 0.03	0.33 ± 0.03	0.05 ± 0.00	0.05 ± 0.00	0.28 ± 0.21	0.09 ± 0.00	0.24 ± 0.11	0.60 ± 0.14
Dandelion	0.13 ± 0.01	0.14 ± 0.01	0.10 ± 0.01	0.11 ± 0.01	0.16 ± 0.04	0.12 ± 0.01	0.20 ± 0.04	0.44 ± 0.05	0.42 ± 0.04
White mulberry leaf	0.14 ± 0.01	0.32 ± 0.02	0.38 ± 0.06	0.15 ± 0.01	0.09 ± 0.00	0.37 ± 0.04	0.18 ± 0.03	0.31 ± 0.08	0.46 ± 0.10
White mulberry fruit	0.32 ± 0.02	0.25 ± 0.03	0.35 ± 0.08	0.39 ± 0.07	0.38 ± 0.04	0.36 ± 0.02	0.26 ± 0.19	0.24 ± 0.01	0.12 ± 0.02

**Table 5:** Two-way analysis of variance for the effects of extraction protocol (MAC, SHA, and UAE), solvent system (water, 70% ethanol, and 96% ethanol), and their interaction on TPC, TFC, TPA, and TAN responses in barberry fruit extracts. The analysis was performed using replicate-level results from three independently prepared extracts per protocol–solvent combination.

Response	Source of Variation	SS	df	MS	F	*p*-Value
TPC	Extraction protocol	8.27	2	4.13	9.54	1.50 × 10^−3^
	Solvent system	38.31	2	19.16	44.20	1.14 × 10^−7^
	Protocol × solvent system	14.06	4	3.51	8.11	6.37 × 10^−4^
	Residual	7.80	18	0.43	—	—
TFC	Extraction protocol	0.49	2	0.24	5.60	1.28 × 10^−2^
	Solvent system	2.04	2	1.02	23.41	9.82 × 10^−6^
	Protocol × solvent system	0.53	4	0.13	3.05	4.39 × 10^−2^
	Residual	0.79	18	0.04	—	—
TPA	Extraction protocol	4.16	2	2.08	6.64	6.90 × 10^−3^
	Solvent system	85.42	2	42.71	136.28	1.34 × 10^−11^
	Protocol × solvent system	15.46	4	3.87	12.33	5.29 × 10^−5^
	Residual	5.64	18	0.31	—	—
TAN	Extraction protocol	0.014	2	0.01	3.00	7.54 × 10^−2^
	Solvent system	0.13	2	0.07	28.5596	2.60 × 10^−6^
	Protocol × solvent system	0.02	4	0.00	1.91	1.53 × 10^−1^
	Residual	0.04	18	0.00	—	—

**Table 6:** Descriptive Pearson correlation coefficients among total phenolic content (TPC), total flavonoid content (TFC), total phenolic acid content (TPAs), and vanillin-reactive tannins (TANs), calculated using pairwise complete condition means. Assay–condition values based on only one valid independent extraction (*n* = 1) were excluded. The number of paired observations is provided in parentheses. The coefficients were not subjected to inferential interpretation.

	TPC	TFC	TPA	TAN
TPC	1			
TFC	0.07 (*n* = 69)	1		
TPA	0.84 (*n* = 70)	0.12 (*n* = 67)	1	
TAN	0.15 (*n* = 72)	0.20 (*n* = 69)	−0.04 (*n* = 70)	1

## Data Availability

The original contributions presented in this study are included in the article/Supplementary Material. Further inquiries can be directed to the corresponding author.

## Supplementary Materials

The following supporting information can be downloaded at: https://www.mdpi.com/article/10.3390/molecules31213993/s1, File S1: Individual extraction-level assay results, condition-level descriptive statistics, complete two-way ANOVA tables, and Tukey HSD pairwise comparisons; File S2: Replicate-level calculation audit, including sample masses, extraction volumes, absorbance values, calibration parameters, dilution corrections, calibration-range verification, back-calculated extract concentrations, and final assay results.
